# Treatment of depression during pregnancy: a protocol for systematic review and meta-analysis

**DOI:** 10.3389/fpsyt.2024.1349816

**Published:** 2024-03-18

**Authors:** Larissa Junkes, Bruno Rabinovici Gherman, Jose Carlos Appolinario, Antonio Egidio Nardi

**Affiliations:** Institute of Psychiatry (IPUB) of Federal University of Rio de Janeiro (UFRJ), Rio de Janeiro, Brazil

**Keywords:** major depressive disorder, pregnancy, anti-depressive agents, drug-related side effects, study protocol, systematic review

## Abstract

**Introduction:**

Major Depressive Disorder (MDD) is a chronic, recurrent, and highly prevalent disease that is associated with significant functional disability. During pregnancy, the prevalence of the disease is approximately 20%, with 12% of these, requiring treatment to avoid important negative consequences for the mother-baby binomial. Risk-benefit assessment of the use of antidepressants during pregnancy is mandatory, in addition to knowledge of the long-term effects of prenatal exposure to these drugs in the offspring. In this study, we will perform an updated systematic review and meta-analysis to explore the treatment of depression during pregnancy, along with its effectiveness, safety, and possible harm to women and children.

**Materials and methods:**

We will search for publications in the following databases: Cochrane Central Register of Controlled Trials (CENTRAL), MEDLINE, Embase, Web of Science, Scopus, Lilacs, and PsycINFO. The reference lists of the included studies will be manually reviewed to identify potentially relevant studies. There will be no restrictions on language or date of publication. Quality assessment of the included studies will be performed independently according to the Cochrane Risk of Bias (RoB2) instrument. To assess the certainty of the findings’ body of evidence, we will use the Grading of Recommendations Assessment, Development and Evaluation (GRADE) methodology. This study aimed to ascertain the efficacy and safety of antidepressants in pregnant women and children.

**Ethics and dissemination:**

Ethical approval was not required as individual patient data were not collected. Dissemination: Plan to publish a systematic review in an open-access medical journal at the end of the process.

**Systematic Review Registration:**

PROSPERO, CRD42023447694.

## Introduction

Major Depressive Disorder (MDD) is one of the most prevalent and widely distributed diseases in the population, affecting more than 300 million people worldwide, and is associated with significant functional impairment in affected individuals (1, 2). During pregnancy, the prevalence of depression is approximately 20%, (3) – and 12% of these require treatment, (4) – which is associated with smoking, maternal malnutrition, alcohol consumption, insufficient weight gain by the pregnant woman, use of other psychoactive substances, and an increased risk of postpartum depression (5). After birth, this percentage is maintained among women in the first six months (4). During this period of intense changes and adaptations, depression, if not properly treated, can have important negative consequences for the mother-baby binomial (6), such as aggravation of the disease (7), premature birth, low birth weight (8), preeclampsia (9), hypertension (10), impaired child development (11), increased risk of psychiatric illnesses in children, and problems with affective attachment (12, 13). In extreme cases, it can increase the risk of infant mortality from neglect, abuse, risky behavior, or homicide (14).

Given the potential risks to the mother and baby, antidepressants are indicated as a first-line treatment for pregnant women with severe depression (15), whereas non-pharmacological interventions are suitable for mild to moderate cases (16). Deciding when to start, switch, or stop antidepressants during pregnancy requires careful weighing of the maternal and infant risks related to treatment and the risks related to the illness itself (17). It is mandatory to compare unfavorable outcomes after intrauterine exposure to antidepressants with those that affect the offspring of women with untreated depression, both to differentiate disease and treatment effects and to provide clinically useful information about the management of women with depression (15).

As studies evaluating antidepressants in pregnancy are generally not randomized, it may be more complex to determine whether the reported harm associated with antidepressants is related to the medication itself, underlying maternal mental illness, genetic differences in risk between women with and without mental illness, or other confounding factors such as exposure to alcohol, tobacco, substance abuse, nutritional deficits, additional drugs, or socioeconomic differences between cohorts (18).

The use of antidepressants during pregnancy is a subject of debate as evidence of adverse fetal and infant outcomes remains inconclusive (19). Studies have found an association between the use of antidepressants by pregnant women and an increased risk of cardiovascular malformation (17, 20, 21), persistent pulmonary hypertension (21, 22), premature birth, low birth weight (23–27) and an increased risk of psychiatric disorders in offspring, including mood disorders, TEA (28), and ADHD (29, 30). There is, to date, a consensus in previous systematic reviews that the increased risk of premature birth and low birth weight is associated with exposure to antidepressants (27); however, the impact of the antidepressants use in the increased risk of congenital anomalies and miscarriage is unclear (15, 23). Another systematic review and meta-analysis observed no significant effect of antidepressant use during pregnancy on the risk of gestational diabetes mellitus (31). French research suggests that antidepressant drugs should be used as a second-line treatment during pregnancy, after first-line psychotherapy, because of the evidence for a significant increase in the risks for the newborn (21).

The prescription of drugs during pregnancy remains a common practice, with antidepressants being the class with the greatest increase in indication over time when compared to other drugs associated with potentially harmful neonatal effects (32). Among pregnant women using antidepressants, 63%-85% use selective serotonin reuptake inhibitors (SSRIs) (17). Among SSRIs, sertraline is one of the most widely used drugs worldwide and is one of the first-choice treatments for depression during pregnancy (33). Despite a significant number of prescriptions, 50% of women discontinued the use of antidepressant medications before or during pregnancy (34, 35).

This systematic review aimed to assess the benefits and harms of antidepressant drug treatment in women with depression during pregnancy, for both mothers and children, during pregnancy, and after delivery.

## Objectives

General: To assess the maternal and child impact of antidepressant drug use during pregnancy.

Specific: To evaluate the response of women with depression to the use of antidepressants and maternal and fetal adverse effects according to the following parameters:

- Depressive symptoms according to the Hamilton Depression Assessment Scale (HAM-D) and Beck Depression Inventory (BDI)
- Risk to self (suicidal and non-suicidal behaviors)
- Child risk (infanticidal behavior, abuse, or neglect)
- Functional capacity according to the Sheehan Disability Scale (SDS)
- Quality of life using validated scales
- Delivery and postpartum parameters: birth weight, gestational age, congenital anomalies, general adverse events, length of post-birth hospital stay, breastfeeding, and mother-infant interaction through validated parameters
- Health outcomes in offspring were measured using well-defined diagnostic criteria and medical diagnosis.

## Method

This systematic review will follow the Preferred Reporting Items for Systematic Reviews and Meta-Analyses (PRISMA). Randomized controlled trials will be searched in the following databases: Cochrane Central Register of Controlled Trials (CENTRAL), MEDLINE, Embase, Web of Science, Scopus, Lilacs, and PsycINFO. The reference lists of the included studies will be manually reviewed to identify potentially relevant studies. There will be no restrictions on language or date of publication. The following sensitive search strategy will be used (MEDLINE): (“Depressive Disorder, Treatment-Resistant”[Mesh] OR (Depressive Disorder*, Treatment Resistant) OR (Disorder*, Treatment-Resistant Depressive) OR (Treatment-Resistant Depressive Disorder*) OR (Refractory Depression) OR (Depression*, Refractory) OR (Refractory Depressions) OR (Therapy-Resistant Depression*) OR (Depression*, Therapy-Resistant) OR (Therapy Resistant Depression) OR (Treatment Resistant Depression*) OR (Depression*, Treatment Resistant) OR (Resistant Depression*, Treatment) OR (“Depressive Disorder, Major”[Mesh] OR (Depressive Disorders, Major) OR (Major Depressive Disorder*) OR (Paraphrenia*, Involutional) OR (Involutional Paraphrenia*) OR (Psychosis, Involutional) OR (Involutional Psychoses) OR (Involutional Psychosis) OR (Psychoses, Involutional) OR (Depression, Involutional) OR (Involutional Depression) OR (Melancholia, Involutional) OR (Involutional Melancholia) AND (“Pregnancy”[Mesh]) OR (Pregnancies OR Gestation) AND (“Antidepressive Agents”[Mesh] OR (Antidepressant Drug*) OR (Drug, Antidepressant) OR (Antidepressant*) OR (Antidepressant Medication) OR (Medication, Antidepressant) OR (Antidepressive Agent) OR (Agent, Antidepressive) OR (Thymoanaleptic*) OR (Thymoleptic*) AND (“Drug-Related Side Effects and Adverse Reactions”[Mesh] OR (Drug-Related Side Effects and Adverse Reaction) OR (Drug Related Side Effects and Adverse Reaction*) OR (Drug Side Effect*) OR (Effects, Drug Side) OR (Side Effect*, Drug) OR (Adverse Drug Reaction*) OR (Drug Reaction*, Adverse) OR (Reactions, Adverse Drug) OR (Adverse Drug Event*) OR (Drug Event*, Adverse) OR (Side Effects of Drugs) OR (Drug Toxicit*) OR (Toxicit*, Drug).

### Selection criteria

Articles must meet the following criteria: (1) the population studied must include women aged 18 years or older, pregnant women of any ethnicity, with a diagnosis of depressive disorder; (2) the intervention using antidepressant drugs administered by any route should be compared with a group of women not using antidepressant drugs or placebo; (3) efficacy – improvement in symptoms of depression as a primary outcome, measured using previously declared validated measurement instruments; (4) patients must have a categorical diagnosis of Major Depressive Disorder (MDD) and Persistent Depressive Disorder (PDD); (5) study design – randomized or open clinical trials, observational non-intervention studies (case-control or cohort designs) and case series; excluded case reports; (6) reference lists: references of articles obtained will be used as a source for investigation of other articles; (7) if there are other interventions combined with antidepressants, they will be evaluated separately; (8) children and adolescents under 18 years of age will be excluded from the study; and (9) incomplete study protocols and animal studies will be excluded; (10) pregnant women diagnosed using DSM5 criteria for bipolar disorder, mood disorder secondary to a general medical condition, mood disorder secondary to substance abuse will be excluded.

### Studies selection

Initially (April/2024) two authors, LJ and BG, will independently screen titles and abstracts, with pre-established criteria, to assess possible eligibility and exclude duplicates, using Rayyan software. Subsequently, LJ and BG will independently assess the full text, according to the defined criteria for inclusion in the review. Any disagreement will be resolved by consensus or consultation with a third party (JCA). The reasons for exclusion will be documented in the report according to the full-text review, and the selection process will be recorded in detail to complete the study selection flowchart of PRISMA (Moher, 2009). In case of multiple publications for the same study, data from the most mature outcomes will be included.

### Data extraction

Two authors will independently extract the data using the form developed for this review. If necessary, authors will be contacted to provide additional relevant or missing information. The data collected will include the following details: authors, year of publication, sociodemographic aspects, study design, intervention (medication, class, and duration of treatment), instruments for assessing the diagnosis of depression, details on the number of participants selected for eligibility, use of intention-to-treat analysis (ITT), outcome data including details of outcome assessment and adverse effects extracted from each of the included studies, detailed interventions, duration of studies and outcomes (with clinical definition and measurement tools), adverse effects, and dropouts.

### Quality assessment

To assess the methodological quality of the included studies, two review authors (LJ and BG) will independently assess the risk of bias using the Cochrane Risk of Bias (Rob2) tool, which is used for systematic reviews that include randomized clinical trials. The instrument is structured into five domains that have ‘signaling questions’, which provide additional information relevant to the assessment of the risk of bias. The response options to the ‘signaling questions’ are: “yes”, “probably yes”, “probably not”, “no”, “no information” and “not applicable”. Definitive “yes” and “no” answers often indicate that robust evidence is available. The “not applicable” option is only available for questions with a non-mandatory answer. Throughout the application of the tool, responses feed an algorithm that determines the risk of bias for each domain: high risk of bias, low risk of bias, or the presence of any concern about bias. In case of a discrepancy in the results between the two authors, a third author (JCA) will be contacted to obtain a consensus. The risk of bias assessment will focus on aspects of participant selection, outcome measurement, and confounding control, encompassing the following domains: randomization, deviation from the intended intervention, missing data, outcome measurement, and reporting of outcomes.

We will evaluate the primary and secondary outcomes. We will judge the risk of bias for each outcome and classify it as having ‘low’, ‘high’ or ‘some concern’ risk of bias. We will judge that incomplete outcome data have a low risk of bias when the number and causes of dropouts are balanced across arms and appear unrelated to the outcome itself. We will assess the outcome selection bias by comparing the outcomes reported in the study protocol with published outcome results. We will use intention-to-treat (ITT) analysis, in which the population will consist of patients who are randomized, use at least one class of antidepressant medication, and are evaluated at least once post-baseline.

### Data analysis

If there are similar studies, and it is possible to group them, a meta-analysis will be performed. To measure the treatment effect, dichotomous outcome data will be analyzed using risk ratio (RR) and 95% confidence interval (CI). Continuous outcome data will be expressed as the mean difference if the outcome is measured in the same manner across studies. The standardized mean difference will be used to combine studies measuring the same outcome, but with different instruments, if possible. We will contact the original investigators and ask for missing data if necessary.

Heterogeneity will be evaluated by visual inspection of the forest plot, together with evaluation of the chi-square test for heterogeneity; it will be considered statistically significant at P<0.1. Studies will also be examined for methodological and clinical heterogeneity (PICO), particularly if statistically significant heterogeneity is identified. For data synthesis, where deemed appropriate, the results from comparable groups across studies will be evaluated using both fixed and random effects. The choice of model for reporting will be guided by careful consideration of the extent of heterogeneity and whether it can be explained, in addition to other factors such as the number and size of included studies. Assessment of the certainty of the body of evidence of the findings will be performed through the Grading of Recommendations Assessment, Development, and Evaluation (GRADE), which evaluates five pillars: risk of bias, imprecision, inconsistency, indirect assessment of outcomes, and publication bias.

## Discussion

The magnitude of the consequences of depression and the progressive increase in its already high prevalence over the years, accompanied by an increase in drug prescriptions, including during the gestational period, makes a detailed study of its treatment possibilities both updated and relevant. Pharmacological collections have expanded, bringing new therapeutic perspectives aimed at reducing the serious impact of this disease. Therefore, improved, precise, and safe treatment is essential for pregnant women with depression.

The management of perinatal depression remains controversial, as there is, so far, no consensus on which drugs are best to use in terms of combined maternal-fetal effectiveness and safety, given the severity of the possible consequences for both the mother and for decreased gestational age, low birthweight, congenital defects, and neurodevelopmental issues for offspring.

Evaluating the existing evidence in the medical literature on the therapeutic alternatives available for the treatment of depression during pregnancy and the role of antidepressants in this subgroup is paramount for guiding clinical practice. So far, the small number of studies containing a group of women with untreated depression as a comparator to reduce confounding – scarce, possibly due to the ethical implications of leaving sick women untreated, at increased risk – is an important limitation in the construction of evidence. There is a need for an accurate estimate of the risks and benefits of both maintenance and discontinuation of antidepressants for pregnant women and children and, in case of the need for discontinuation, which is the most appropriate therapeutic alternative to continue the treatment, to develop evidence-based clinical guidelines to support the rational treatment of depression during pregnancy.

## Ethics statement

Ethical approval was not required as individual patient data were not collected. Dissemination: Plan to publish a systematic review in an open-access medical journal at the end of the process.

## Author contributions

LJ: Conceptualization, Formal analysis, Investigation, Methodology, Project administration, Software, Writing – original draft. BG: Data curation, Formal analysis, Investigation, Writing – review & editing. JA: Data curation, Formal analysis, Writing – review & editing. AN: Conceptualization, Data curation, Formal analysis, Methodology, Project administration, Supervision, Writing – review & editing, Resources.
